# Multicenter retrospective study on effectiveness, reported side effects, and cognitive outcomes of SSRIs in 22q11.2 deletion syndrome

**DOI:** 10.21203/rs.3.rs-8286229/v1

**Published:** 2025-12-11

**Authors:** Caren Latrèche, Valentina Mancini, Marija Dvojakovska, Leila Kushan, Fatouma Mchangama, Feryal Tair, Tal Cohen, Jeltje Spapens, Lieke Reijn, Covadonga M. Díaz-Caneja, Hayford Acheampong, Lotte Troch, Elfi Vergaelen, Annick Vogels, Ann Swillen, Claudia Vingerhoets, Erik Boot, Celso Arango, Fleur Velders, Ania Fiksinski, Therese van Amelsvoort, Doron Gothelf, Carrie E. Bearden, Boris Chaumette, Maude Schneider, Stephan Eliez

**Affiliations:** University of Geneva; Oxford University, University of Oxford; Université Paris Cité, UFR de médecine; University of California; Hôpital Sainte Anne; Hôpital Sainte Anne; Sheba Hospital; Maastricht University; Wilhelmina Children’s Hospital; Hospital General Universitario Gregorio Marañón, Universidad Complutense; Hospital General Universitario Gregorio Marañón, Universidad Complutense; University Hospital Leuven; University Hospital Leuven; University Hospital Leuven; University Hospital Leuven; Maastricht University; ’s Heeren Loo Zorggroep; Ciber del Área de Salud Mental (CIBERSAM), Instituto de Salud Carlos III; University Medical Center Utrecht; Wilhelmina Children’s Hospital; Maastricht University; Sheba Hospital; University of California; Hôpital Sainte Anne; University of Geneva; University of Geneva

**Keywords:** 22q11.2 deletion syndrome, Selective serotonin reuptake inhibitors (SSRIs), IQ, Cognitive trajectories, Multicenter retrospective study

## Abstract

22q11.2 deletion syndrome (22q11DS) markedly increases risk of psychiatric disorders, including anxiety and mood disorders, and is associated with a spectrum of cognitive impairment, from borderline functioning to intellectual disability, with cognitive decline frequently reported. Despite widespread use of selective serotonin reuptake inhibitors (SSRIs) in 22q11DS, evidence regarding their safety, effectiveness, and potential effects on cognitive trajectories remains limited. We conducted a retrospective, observational multicenter study across nine international sites, including cross-sectional and longitudinal parts. In the cross-sectional part, 190 SSRI-treated participants with 22q11DS (6–56 years) were included to characterize indication, perceived effectiveness, and side effects. In the longitudinal part, intellectual quotient (IQ) trajectories were compared between 101 SSRI-treated and 214 SSRI-untreated participants (4–34 years) using mixed-models regression analyses. SSRIs were mainly prescribed for mood and/or anxiety disorders (91%) and were mostly effective or very effective (71%), with minimal reported side effects for the majority (75%). SSRI-treated participants exhibited stable or modestly improving IQ trajectories, compared with SSRI-untreated participants. Combined SSRI + psychostimulant treatment was associated with the largest improvements. Treatment duration, but not dosage, was positively associated with IQ change. SSRIs appear safe and effective for mood and anxiety disorders in 22q11DS and are associated with a modest improvement in cognitive functioning, particularly with sustained use. Concomitant psychostimulant treatment was linked to the greatest cognitive gains. These findings highlight the importance of screening and treatment of psychiatric symptoms to optimize long-term cognitive outcomes. Controlled prospective studies are needed to confirm the findings and determine underlying mechanisms.

## Introduction

22q11.2 deletion syndrome (22q11DS) is a copy number variant resulting from a hemizygous deletion on the long arm of chromosome 22 [1]. 22q11DS is associated with elevated risk for a broad range of psychiatric conditions, including attention-deficit/hyperactivity disorder (ADHD), anxiety disorders, obsessive-compulsive disorder (OCD), depression, and schizophrenia [2]. Despite the high psychiatric burden, there are currently no pharmacological treatments specific to 22q11DS. Individuals with this condition and comorbid psychiatric diagnoses are typically managed using treatment strategies developed for the general population [3].

Current evidence supports the use of standard pharmacological treatments in 22q11DS for psychiatric disorders [4, 5]. For example, psychostimulants (PS) such as methylphenidate appear to have comparable efficacy and tolerability for treating ADHD symptoms in individuals with 22q11DS as in non-deleted populations, as demonstrated by one open-label study [6] and two clinical trials [7, 8]. Antipsychotics have also been widely studied for managing psychotic symptoms in 22q11DS, though deletion carriers may be more susceptible to side effects [9–11].

To date, evidence supporting the use of selective serotonin reuptake inhibitors (SSRIs) in 22q11DS remains limited. Available data consist primarily of one retrospective study and a case series [12, 13]. Nevertheless, SSRIs are commonly prescribed to treat anxiety, depression, and OCD in this population. In addition, data from studies in non-deleted patients with depression and at psychosis risk suggest that SSRIs may exert positive effects on cognitive function [14–16].

The potential cognitive effects of SSRIs require investigation in 22q11DS, considering the wide range of cognitive deficits [17–19]. Intelligence quotient (IQ) is the most used metric to assess cognitive functioning in individuals with 22q11DS. Numerous studies have reported lower IQ scores in individuals with 22q11DS compared to the general population, with the full-scale IQ (FSIQ) distribution shifted leftward by approximately two standard deviations (mean FSIQ ≈ 72) [20, 21]). Longitudinal data indicate that individuals with 22q11DS often experience a decline in IQ over time, particularly during adolescence [22]. This decline is thought to reflect a slower pace in cognitive development compared to typically developing peers, and in adulthood, may indicate an accelerated loss of cognitive capacity relative to the general population [20]. Such findings underscore the vulnerability of this population to progressive cognitive difficulties.

Concerning a potential positive effect on cognitive function of SSRIs in 22q11DS, preliminary observational studies have suggested that long-term treatment with SSRIs may attenuate cognitive decline. One longitudinal study [23] reported improved general cognitive performance with psychiatric medication, although the effect was nonspecific, including medications beyond SSRIs. Another recent longitudinal study [24] specifically examined 36 individuals with 22q11DS receiving SSRI treatment. Interestingly, they did not exhibit the expected longitudinal drop in IQ, raising the possibility that serotonergic modulation may exert neuroprotective effects in this population by enhancing synaptic plasticity. However, these initial findings need to be extended in larger, well-characterized cohorts to determine their robustness and clinical relevance.

This multicenter study included participants with 22q11DS, treated and untreated with SSRIs, recruited across nine international centers. It aimed to address existing gaps by first evaluating the safety and effectiveness of SSRIs in a cross-sectional sample of 190 treated individuals with 22q11DS. Building on preliminary findings from a smaller single-center cohort [24], this follow-up study revisits and extends the initial longitudinal sample to 314 SSRI-treated and SSRI-untreated individuals to examine the associations between SSRI treatment and IQ trajectories. In line with previous studies [23, 24], we expect higher IQ scores in SSRI-treated participants compared to SSRI-untreated participants. Additionally, given the high prevalence of ADHD in 22q11DS (35–40%) [2] and the procognitive effects of psychostimulants [25], we explored potential associations between combined SSRI + PS treatment and IQ trajectories.

## Methods

### Study design

This retrospective, observational multicenter study was conducted across nine centers in Switzerland, France, Belgium, The Netherlands, Spain, Israel and the USA. The study included patient data collected between August 2005 and June 2025.

## Ethical considerations

The study was undertaken in accordance with the Declaration of Helsinki. Ethical approval was obtained from the local ethics committees of all participating centers. Written informed consent for data use was obtained from each participant and, when applicable, their legal caregiver, in accordance with local regulations and ethical requirements.

Each center completed a standardized spreadsheet template with de-identified clinical data. A unique study ID was assigned to each participant to anonymize records and identify potential duplicates across sites. Each completed spreadsheet was transferred to the coordinating center (Geneva). The data were cleaned and pooled into a single dataset for analysis.

## Cross-sectional analysis

### Participants

The cross-sectional study included 190 participants (101 males) with 22q11DS (Table 1). The participants’ age ranged from 6 to 56 years (*M* = 23.8, *SD* = 9.7). Inclusion criteria were (1) a genetically confirmed 22q11.2 deletion, and (2) current or past treatment with SSRIs, with information on the specific SSRI prescribed.

## Materials

We collected information on the type of SSRI prescribed, indication, perceived effectiveness, and reported side effects. Data completeness varied across sites due to differences in reporting practices. Information on indication, perceived effectiveness, and side effects was available for 70.5%, 45.3%, and 21.1% of the sample, respectively.

First, indications for SSRI prescription were reported based on the condition treated, as documented by each center. Second, the perceived effectiveness of SSRIs was assessed on a 5-point scale (0 = ineffective, 1 = slightly effective, 2 = moderately effective, 3 = effective, 4 = very effective). Ratings were provided either by clinicians or reported by participants/caregivers depending on center protocol. Thirdly, centers reported side effects using a predefined list of common SSRI-related side effects (0 = none; 1 = drowsiness/sleepiness; 2 = sexual dysfunction; 3 = weight gain; 4 = dry mouth; 5 = insomnia; 6 = fatigue; 7 = nausea; 8 = dizziness; 9 = tremors/shaking hands; 10 = other).

### Statistical analyses

Descriptive statistics were performed using Microsoft Excel (version 365). Pie charts were generated to visually represent the variables related to SSRI use, with the data presented as percentages.

## Longitudinal analysis

### Participants

To examine the potential associations between SSRI treatment and IQ trajectories, we used longitudinal IQ data available from six of the nine centers to compare trajectories between SSRI-treated and SSRI-untreated participants.

Inclusion criteria for the whole sample were: (1) a genetically confirmed 22q11.2 deletion; and (2) the ability to complete a standardized cognitive assessment. Additional inclusion criteria for the SSRI-treated group were: (1) a duration of SSRI treatment ≥ 1 month; and (2) either one IQ assessment before and at least one after SSRI onset, or one IQ assessment during SSRI treatment (see Statistical analyses). Exclusion criteria for the SSRI-treated group were: (1) unknown SSRI start date or treatment duration (*N* = 7); and (2) no IQ assessment (*N* = 20). For the SSRI-untreated group, inclusion required at least one IQ assessment without SSRI treatment, and the exclusion criterion was history of SSRI treatment (*N* = 1).

The final sample size included 314 participants with 22q11DS, including 101 treated with SSRIs (see Table 2 for the distribution across centers). The participants’ age ranged from 4 to 34 years. The upper age limit was set to ensure reliable group comparisons, given the limited sample of adults aged 35 or older (*N* = 25, 11 SSRI-treated and 14 SSRI-untreated). The SSRI-treated and SSRI-untreated groups were comparable for age, sex, FSIQ, PIQ, and VIQ at their first time-point (TP1; Table S1). Across the 314 participants, a total of 659 TPs were acquired, with 67% belonging to the SSRI-untreated group. The mean number of TPs per participant was comparable between SSRI-treated and SSRI-untreated groups (*M* = 2.17, *SD* = 0.96 vs. *M* = 2.07, *SD* = 1.14, respectively; *U* = 9829, *p* = 0.174). The number of TPs ranged from 1 to 6 and were spaced an average of 3.67 years apart (*SD* = 2.58), with no significant differences between groups in assessment intervals (*U* = 12115, *p* = 0.146). From our final sample of 314 participants, a subset from the Geneva center (*N* = 57; 36 SSRI-treated and 21 SSRI-untreated) overlapped with those included in our preliminary study [24].

In addition, we created three groups to examine the role of SSRIs and PS on FSIQ outcomes. The SSRI-treated group was divided into two subgroups based on concomitant PS use (SSRI + PS: *N* = 25; SSRI + noPS: *N* = 44). As 98% of the SSRI-untreated group was also not receiving PS treatment, we included an independent longitudinal sample of participants treated with PS (noSSRI + PS, *N* = 34) from the Geneva cohort. The three subgroups were comparable for age, sex, but not FSIQ, at their TP1 (Table S2). An additional analysis comparing four groups (SSRI + PS, SSRI + noPS, noSSRI + PS, noSSRI + noPS) is provided in Figure S1.

### Cognitive assessment

Intellectual functioning was assessed at each TP using a Wechsler Intelligence Scale. Due to variability across centers, 11 different versions were used for the 659 assessments (Table S3).

For young children (ages 2 years 6 months to 7 years 7 months), the WPPSI-III, WPPSI-R, or WPPSI-IV was administered [26, 27]. Children aged 6–16 years 11 months were assessed using the WISC-R, WISC-III, WISC-IV, or WISC-V [28–31]. For participants aged 17 years and older, the WAIS-III, or WAIS-IV was used [32, 33]. In addition, two abbreviated scale versions were administered to a subsample of children and adults (WASI-I and WASI-II[34, 35]). FSIQ scores were available for all 314 participants. Verbal IQ (VIQ) was available for 213 participants (450 TPs) and Performance IQ (PIQ) for 195 participants (424 TPs). Missing VIQ and PIQ data were either due to the use of Wechsler versions that did not include these indices or to centers not computing or reporting them.

### Statistical analyses

First, descriptive statistics were performed using Graphpad Prism 9.5.1 (GraphPad Software, San Diego, CA, USA) to allow age, sex, and IQ comparisons between SSRI-treated and SSRI-untreated groups. As in our preliminary study [24], we conducted mixed-model regression analyses using MATLAB R2021a (Mathworks, Natick, MA, USA) to analyze longitudinal IQ data, allowing comparability across analyses. This approach is well suited for handling repeated measures with varying numbers of TPs, and inconstant time interval and age distribution [36]. Importantly, it allows inclusion of participants with multiple IQ assessments as well as those with only a single assessment, ensuring that all available data contribute to the analysis. Linear mixed-effects models were fitted using the *nlmefit* function, with age and diagnosis as fixed effects and individual variation as random effects, to model changes in IQ with age, consistent with our preliminary study [24]. Group differences in IQ trajectories were assessed by comparing full and reduced models using likelihood ratio tests. Where relevant, the age at which developmental change shifted direction (inflection point) was also estimated. Sex, center, and Wechsler version were added as covariates. In addition to age, we modelled changes in IQ with TPs, to allow for a more standardized analysis of the effects of SSRI, as age at treatment onset varies across centers and participants. In this case, we entered age as an additional covariate. We restricted the maximum number of TPs to 4 (instead of 6) for FSIQ, as only eight participants had a 5th or 6th TP. The same procedure was applied for VIQ and PIQ.

Finally, correlational analyses were conducted in the SSRI-treated group including only participants with at least two TPs, using GraphPad Prism 9.5.1. Depending on data distribution (assessed with the Shapiro-Wilk test), either Pearson’s or Spearman’s correlations were used, followed by false discovery rate (FDR) correction for multiple comparisons. We examined associations between ΔIQ (difference between the last and first IQ scores) and individual characteristics, including age at SSRI treatment onset, dosage (fluoxetine equivalents normalized by body weight in kg), treatment duration, and baseline IQ. Analyses were performed separately for FSIQ (*N* = 77), PIQ (*N* = 51), and VIQ (*N* = 64). Due to missing body weight data, sample sizes for dosage analyses were reduced to *N* = 57 for FSIQ and *N* = 45 for both PIQ and VIQ.

## Results

### Cross-sectional analysis

**Figure 1:**
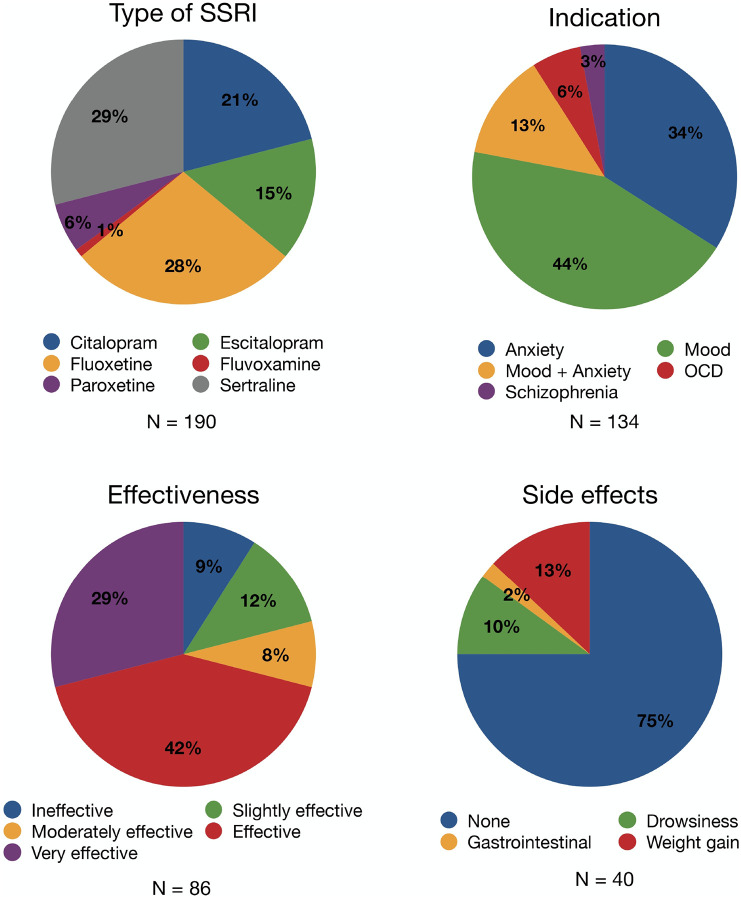
Distribution of type of SSRI prescribed, indications for prescription, perceived effectiveness and reported side effects in individuals with 22q11DS

The findings characterizing SSRI use in 22q11DS are shown in Fig. 1. The most prescribed SSRIs in 22q11DS were sertraline (29%) and fluoxetine (28%), followed by citalopram (21%), escitalopram (15%), paroxetine (6%), and fluvoxamine (1%). Regarding the indications for prescription, mood disorders were the most frequent (44%), followed by anxiety disorders (34%), combined mood and anxiety disorders (13%), obsessive-compulsive disorder (6%), and schizophrenia (3%). For most participants with data (*N* = 40), no side effects were reported (75%), while smaller proportions reported weight gain (13%), drowsiness/sleepiness (10%), and gastrointestinal symptoms (2%). In terms of perceived effectiveness (available for *N* = 86), most participants, caregivers, and clinicians rated SSRI treatment as effective (42%) or very effective (29%), followed by slightly effective (12%), ineffective (9%), and moderately effective (8%).

### Longitudinal analyses

**Figure 2:**
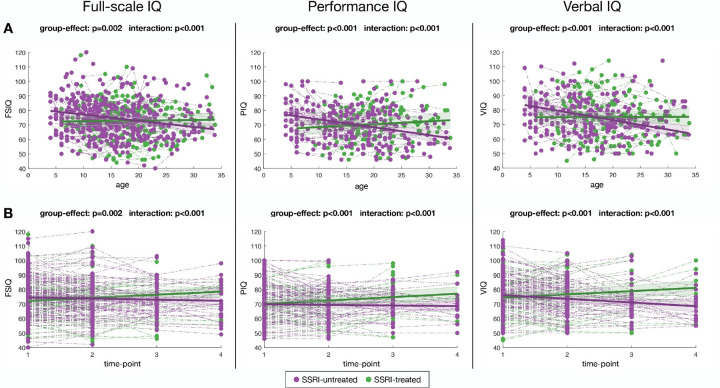
Developmental trajectories of full-scale IQ (FSIQ), performance IQ (PIQ), and verbal IQ (VIQ) in SSRI-treated and SSRI-untreated participants across age (**A**) and time-points (**B**)

**Figure 3:**
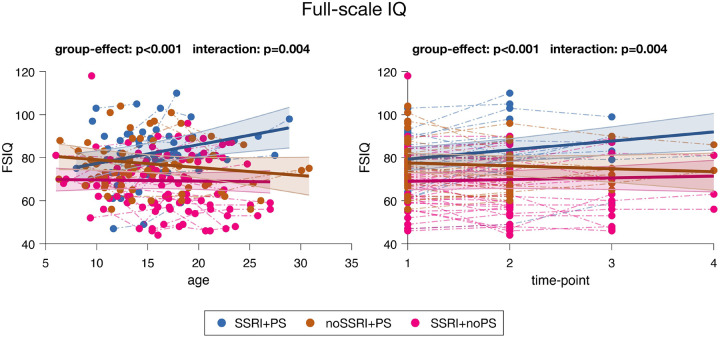
Developmental trajectories of full-scale IQ (FSIQ) into three subgroups of participants: (1) treated with both SSRIs and PS (SSRI+PS); (2) untreated with SSRIs but treated with PS (noSSRI+PS); and (3) treated with SSRIs but untreated with PS (SSRI+noPS), across age and time-points

Developmental trajectories of FSIQ, PIQ, and VIQ were compared between SSRI-treated vs. SSRI-untreated participants to examine the effect of SSRIs on IQ trajectories (Figs. 2 and 3, Table S4).

First, when modelling IQ scores as a function of age (Fig. 2a), we found significant group effect and group × age interaction (*p* = 0.002 and *p* < 0.001, respectively). Participants treated with SSRIs displayed a lower FSIQ at baseline compared to those untreated (71.99 vs. 81.32, respectively). Moreover, the SSRI-treated group exhibited a stable or slightly increasing trajectory, while the SSRI-untreated group demonstrated a relative FSIQ decline over development (0.05 vs. −0.43 points per year, respectively). A similar pattern was observed for PIQ (*p* < 0.001 and *p* < 0.001, for group effect and interaction), with an average gain of 0.20 points per year in the SSRI-treated group and average loss of 0.54 points in the SSRI-untreated group. Comparable findings are shown for VIQ, with significant group effect (*p* < 0.001) and interaction with age (*p* < 0.001). The SSRI-treated group maintained stable VIQ over time (0.01 points per year), while the SSRI-untreated group exhibited decreased VIQ (−0.65 points per year). Interestingly, the divergence between SSRI-treated and SSRI-untreated trajectories emerged a few years after the initiation of SSRI treatment, between late adolescence (around 17 years, for PIQ and VIQ) and early adulthood (around 20 years for FSIQ), given an average age at treatment onset of 16.7 years (*SD* = 6.20).

Second, we examined IQ trajectories across four TPs (TP1 to TP4; Fig. 2b), spaced on average 3.67 (*SD* = 2.58) years apart (see Table S5 for mean ages and age ranges at each TP). At TP1, SSRI-treated participants again exhibited lower baseline scores in FSIQ, PIQ, and VIQ (*p* < 0.001) compared to untreated participants. However, they showed significant improvement over subsequent TPs (TP2-TP4) for all three IQ measures (*p* < 0.001). The SSRI-treated group gained an average of 2.13, 2.35, and 2.24 points per interval between TPs for FSIQ, PIQ, and VIQ, respectively (ΔIQ ranges are provided in Table S6). In contrast, the SSRI-untreated group displayed a cumulative loss of − 0.80, − 0.39, and − 2.54 points, respectively.

Furthermore, we compared FSIQ trajectories across three subgroups based on SSRI and PS use (i.e. SSRI + PS, noSSRI + PS, SSRI + noPS; Fig. 3 and Table S7). We found significant group effect and interaction, both in terms of age and TPs (p < 0.001 and p = 0.004, respectively). Participants treated with both SSRIs and PS exhibited an average FSIQ increase of 0.88 points per year, corresponding to 4.25 points gained per interval between TPs. The SSRI + noPS subgroup displayed a relatively stable trajectory over the years (−0.05 points per year), or an increase when measured per interval between assessments (0.80 points). In contrast, the noSSRI + PS subgroup shows a decrease in FSIQ scores over time (−0.37 points per year and − 1.35 points per interval between TPs), similar to the SSRI-untreated group. Additional mixed-model analyses were performed to compare our three subgroups two-by-two. Significant group effect and interaction were observed between SSRI + PS vs. noSSRI + PS and SSRI + PS vs. SSRI + noPS, while only a group effect was found between noSSRI + PS vs. SSRI + noPS (Figure S2 and Table S8).

### Correlational analyses

Correlational results are shown in Table S9. Spearman’s correlation analyses revealed a significant positive association between SSRI treatment duration and change in FSIQ (ΔFSIQ; *r* = 0.294, adjusted *p* = 0.036), and a trend for ΔPIQ (*r* = 0.310, adjusted *p* = 0.068). No significant correlations were found between treatment duration and ΔVIQ (*r* = 0.204, adjusted *p* = 0.212). Regarding age at SSRI treatment onset, no significant correlations were found with ΔFSIQ or ΔPIQ. However, a negative association was observed with ΔVIQ, but it did not survive FDR correction (*r* = − 0.281, adjusted *p* = 0.096). Dosage (expressed as fluoxetine-equivalent mg/kg) was not significantly associated with any IQ variables. Finally, correlations between baseline IQ scores and ΔIQ scores only yielded a negative trend-level association between baseline PIQ and ΔPIQ (*r* = − 0.297, adjusted *p* = 0.068).

## Discussion

This is the first multicenter study in 22q11DS to assess the perceived effectiveness and side effects of SSRI treatment, and its associations with IQ trajectories, using a retrospective, naturalistic design.

### Side effects and effectiveness of SSRIs

Our cross-sectional findings first show that a variety of SSRIs were prescribed in individuals with 22q11DS, primarily for mood and anxiety disorders. For 75% of treated individuals with data, no side effects were documented, suggesting an overall favorable safety profile. These observations are consistent with findings in non-22q11DS populations, where SSRIs are generally well tolerated [37–39]. Regarding effectiveness, our findings revisit and extend previous work in 22q11DS [24]. We found a response rate of 71% (effective to very effective), corroborating earlier findings in a sample of 16 individuals with 22q11DS with depressive and anxiety disorders (58–70%) [12]. These proportions are slightly higher compared to those reported in non-22q11DS patients (40–60%) [39–42]. Together, these findings suggest a favorable risk-benefit profile for SSRI treatment in 22q11DS.

### IQ outcomes following SSRI treatment

Our longitudinal analysis revealed distinct IQ trajectories between participants treated with SSRIs and those who remained untreated, in line with our previous findings in a partly overlapping, smaller sample [24]. Participants receiving SSRI treatment exhibited a slight increase in IQ over an average 3-year period, ranging from + 2.13 to + 2.35 points across VIQ, PIQ, and FSIQ. While the magnitude of these gains is modest, they are meaningful in the context of the untreated group, which showed reductions in IQ (−0.39 to −2.54 points across the same measures) over the same interval, consistent with the typical trajectory of cognitive decline in 22q11DS [22]. Importantly, the mean age at SSRIs initiation preceded the divergence in FSIQ trajectories, and a younger age at SSRI treatment onset tended to be associated with higher VIQ. This is consistent with prior work [24], suggesting a possible role of earlier treatment in IQ outcomes. Moreover, a significant positive association was observed between SSRI treatment duration and higher FSIQ, that may support a cumulative effect of serotonergic modulation. No significant associations were found with SSRI dosage, suggesting that timing and duration, rather than dose intensity, are more influential in determining IQ outcomes.

Some of the observed cognitive improvements may reflect indirect clinical effects of SSRIs. By reducing anxiety and depressive symptoms, SSRIs could improve cognitive test performance. Conversely, untreated psychiatric symptoms in the SSRI-untreated group may have suppressed IQ scores. We cannot exclude that part of the cognitive gains arises from improved clinical state. Future studies integrating standardized measures of mood and anxiety alongside cognitive assessments are needed to disentangle direct versus indirect effects of SSRIs.

### IQ outcomes following combined SSRIs and PS treatment

We further investigated whether combining SSRIs with PS, which are commonly prescribed in 22q11DS, influences IQ trajectories. Individuals receiving both SSRIs and PS demonstrated greater cognitive improvements than those who received only one or neither treatment. Cognitive function in 22q11DS has been linked to serotonergic, noradrenergic, and dopaminergic systems. A previous study in adults with 22q11DS reported positive associations between FSIQ and urinary serotonin (5-HT), dopamine, and norepinephrine metabolite concentrations [43], suggesting that alterations across these neurotransmitter systems may contribute to cognitive variability.

From a clinical perspective, the addition of PS might contribute to alleviating attentional difficulties, enhancing motivation and engagement. However, this alone does not explain the observed synergistic effect of SSRIs and PS. The potential synergy between SSRIs and PS may reflect serotonergic–dopaminergic interactions [44]. Preclinical evidence in stressed wild-type mice indicates that D1 receptor agonists can potentiate the effects of SSRIs on hippocampal neurogenesis and behavioral outcomes [45]. Although PS do not act directly as D1 receptor agonists, they increase synaptic dopamine levels by inhibiting dopamine reuptake, which may indirectly augment SSRI efficacy via increased stimulation of D1 receptors. This mechanism may provide a plausible explanation for the observed cognitive benefits of combined SSRI and PS treatment. Alternatively, the observed synergy may reflect broader serotonergic–dopaminergic interactions that jointly regulate reinforcement learning and cognitive control [46]. Dopamine facilitates behavioral activation and reward learning, whereas serotonin promotes behavioral inhibition and learning from negative outcomes [46, 47]. Coordinated modulation of these systems may rebalance activation–inhibition dynamics, enhancing cognitive flexibility and overall cognitive performance.

### Clinical implications

Our findings suggest the effectiveness and limited side effects of SSRIs in treating mood and anxiety disorders in 22q11DS, which is important as anxiety disorders affect approximately one-third of individuals (prevalence of 31%) [2]. In addition, our findings showed that combined treatment with SSRI and PS was associated with the largest beneficial effects on IQ trajectories. Specifically, methylphenidate has been reported as well-tolerated and effective for addressing attentional difficulties, which are overrepresented in this population [7, 8]. Importantly, given the increased prevalence of cardiovascular anomalies, individuals with 22q11DS are at increased risk of cardiac side effects [48, 49]. ECG screening to monitor QTc interval and detect potential arrhythmia should be considered when prescribing SSRI + PS. In this context, such monitoring can also provide reassurance to families and therapists and facilitate the safe use of combined medications, which is often necessary given the frequent co-occurrence of anxiety, mood and attentional difficulties. Therefore, our results highlight the importance of early screening for anxiety, mood, and ADHD symptoms in individuals with 22q11DS, as introducing adequate treatment may help stabilize or even improve cognitive functioning. Our findings further raise questions about the potential cognitive benefits of preventive treatment in subthreshold or asymptomatic individuals with 22q11DS.

### Strengths and limitations

Major strengths of this study are its longitudinal, multicenter design and the relatively large treated and untreated 22q11DS samples, which allowed us to characterize SSRI treatment and investigate IQ trajectories in a large age range.

However, several limitations should be noted. First, the retrospective design and multicenter nature led to missing and heterogeneous data, particularly regarding reports of perceived effectiveness (45% of participants) and side effects (21%). Furthermore, sources of information varied across sites (e.g., questionnaire lled by participants, clinical-rated impression scales completed by clinicians). Discontinuation or switching of SSRIs was not systematically assessed, potentially excluding individuals who interrupted medication early due to side effects. These factors could partly explain the low rates of side effects and high rates of perceived effectiveness. Second, multiple versions of the Wechsler scales were used across sites. Site and scale version were therefore added as covariates to limit bias in mixed-model regression analyses. Third, the retrospective design prevented us from controlling for potential confounding factors, such as concomitant psychotropic medication. Regarding our exploratory analyses on SSRI and PS, dividing the SSRI-treated group into three subgroups reduced statistical power, particularly for the SSRI + PS subgroup (*N* = 25). Baseline differences in FSIQ between subgroups may have influenced longitudinal trajectories, and we cannot exclude that these differences contributed to the observed outcomes. Altogether, these findings underscore the need for future prospective trials that can address the challenges of conducting randomized double-blind studies in a rare condition such as 22q11DS [50]. Finally, this study relied exclusively on behavioral outcome measures.

## Conclusion

This multicenter study suggests that SSRIs are effective for treating mood and anxiety disorders in individuals with 22q11DS, with limited side effects. SSRI treatment was associated with modest IQ improvements over time, particularly when maintained long-term. Combining SSRIs with PS further enhanced cognitive outcomes, likely through synergistic serotonin–dopamine effects. These findings underscore the importance of early screening for anxiety, mood, and ADHD symptoms to enable timely intervention that may improve cognitive development and reduce multimorbid psychiatric risks. However, future prospective and well-powered studies are needed to confirm the findings.

## Supplementary Material

Supplementary Files

This is a list of supplementary files associated with this preprint. Click to download.

- SupplementaryMaterial.docx

## Figures and Tables

**Table 1:** Number of participants with 22q11DS treated with SSRIs included across sites in Switzerland, France, the USA, Israel, the Netherlands, Spain, and Belgium.

Site	Country	Principal investigators	N	Age range (years)
Geneva	Switzerland	S. Eliez, M. Schneider	54	9–27
Paris	France	B. Chaumette	31	13–36
Los Angeles	USA	C. Bearden	28	9–39
Tel Aviv	Israel	D. Gothelf	19	26–53
Maastricht	The Netherlands	T. van Amelsvoort	18	18–56
Utrecht	The Netherlands	A. Fiksinski, F. Velders	12	14–27
Madrid	Spain	C. Arango	11	9–25
Leuven	Belgium	A. Swillen	9	6–28
Noordwijk	The Netherlands	E. Boot, C. Vingerhoets	8	20–52
Total	9		190	6–56

**Table 2:** Number of participants and IQ assessments (time-points; TPs) by site and SSRI treatment status.

	N			Time-points		
Site	SSRI-treated	SSRI-untreated	Total N	SSRI-treated	SSRI-untreated	Total TPs
Geneva	52	21	73	140	63	203
Maastricht	10	76	86	15	94	109
Leuven	5	43	48	9	110	119
Los Angeles	26	55	81	42	138	180
Madrid	4	18	22	8	35	43
Utrecht	4	0	4	5	0	5
Total	101	213	314	219	440	659

## Data Availability

The raw data supporting the conclusions of this article will be made available by the authors, on a reasonable request.
